# Supplementary material for: Mechanistic insights into alcohol-induced DNA crosslink repair by Slx4-Xpf-Ercc1 nuclease complex in the Fanconi anaemia pathway
Source: Commun Biol. 2025 Sep 26;8:1374. doi: 10.1038/s42003-025-08769-3 (PMC12475018; doi:10.1038/s42003-025-08769-3)

Mechanistic Insights into Alcohol-Induced DNA Crosslink Repair by Slx4-Xpf-Ercc1 Nuclease Complex in the Fanconi Anaemia Pathway

Jana Havlikova[1,2], Milan Dejmek[1], Andrea Huskova[1], Anthony Allan[3], Evzen Boura[1], Radim Nencka[1], Jan Silhan[1,*]

[1] Institute of Organic Chemistry and Biochemistry of the Czech Academy of Sciences, Flemingovo namesti 2, 160 00 Prague, Czech Republic

[2] Charles University, First Faculty of Medicine, Katerinska 32, 121 08 Prague, Czech Republic

[3] Charles University, Faculty of Science, Albertov 6, 128 00 Prague, Czech Republic

*To whom correspondence should be addressed. Tel: +420 220 183 464; Email: silhan@uochb.cas.cz

Supplementary Figure S1: ICL substrates.

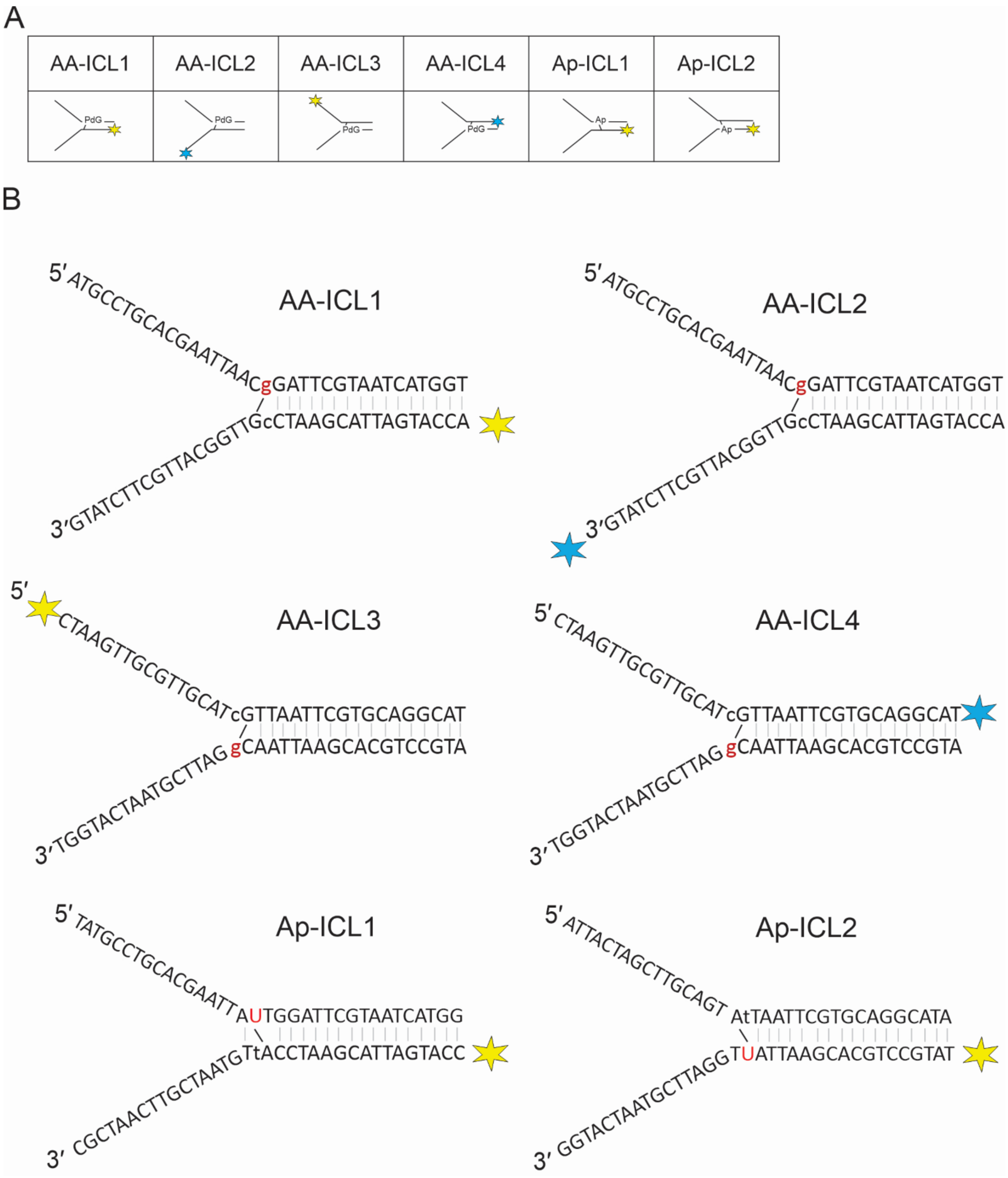

(A) Representation and (B) sequences of prepared ICL used in this study. g corresponds to (*R*)-α-CH3-γ-OH-1,N^2-propano-2’-deoxyguanosine (PdG). The Ap-ICL is formed by a covalent linkage between an abasic (Ap) site generated by uracil excision by UDG and an adenine residue on the opposite strand. This adenine is located adjacent to an orphaned thymine (dT) opposite the Ap site, specifically positioned 5′ to the thymine (marked as a line between dU and A in opposite strand).

Supplementary Figure S2: Preparation of ICL.

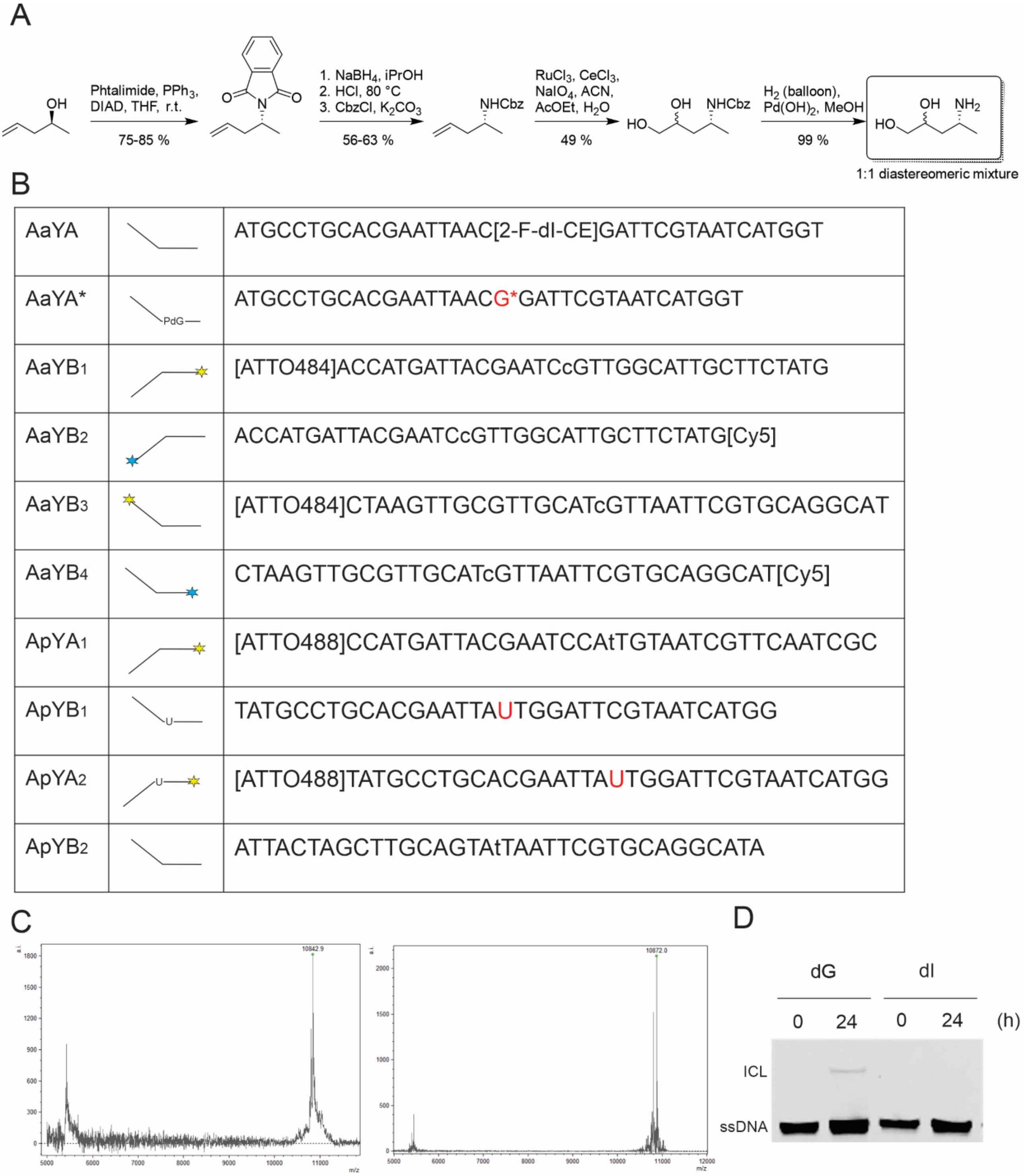

(A) Scheme of synthesis of (4*R*)-4-aminopentane-1,2-diol. (B) Sequences of DNA oligonucleotides used in this study, where G* corresponds to (*R*)-α-CH3-γ-OH-1,N^2-propano-2'-deoxyguanosine (PdG). The star's colour refers to the fluorescent dye used (yellow - ATTO488, blue - Cyanin5). (C) Representation of prepared ICL used in this study. (D) MALDI-MS spectrum of the modified oligonucleotide before (10873,7) and after oxidation (10845,1). (E) Denaturing PAGE gel shows the formation of AA-ICL in the presence of deoxyguanosine (dG), however, in the presence of deoxyinosine (dI), no AA-ICL is formed.

Supplementary Figure S3: Complementary cleavage mechanism of AA-ICL (fluorescently labelled on the bottom strand) by SXE nuclease.

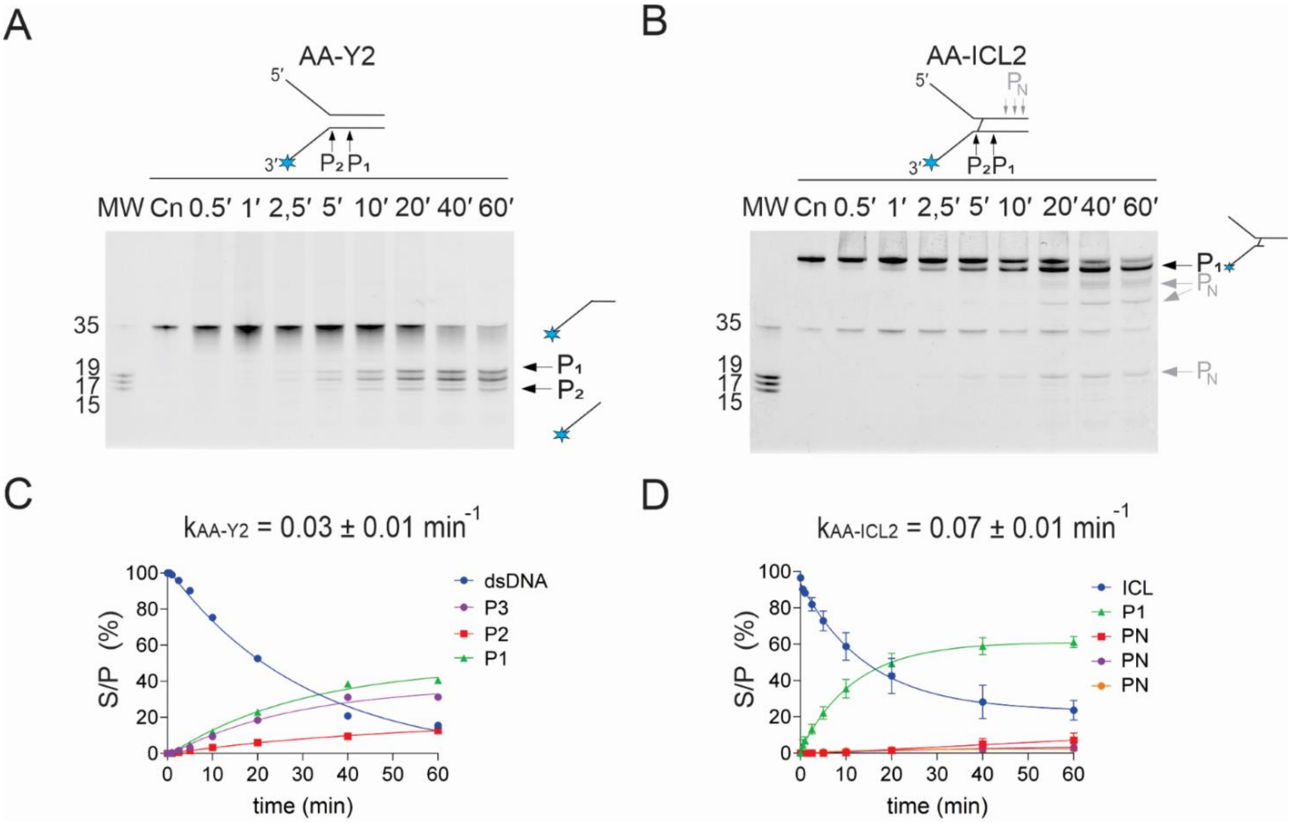

(A) SXE nuclease assay (quantitative) with non-crosslinked **Y2** substrate show the formation of cleavage products P_1 (short 15-nt product) and P_2 (19-nt product) and third 17-nt product. P_2, from the second incision of the non-crosslinked control corresponding to the same products as the incision of AA-Y1 and AA-ICL1. **(B)** SXE nuclease assay (qualitative) with crosslinked **AA-ICL2** substrate. The products include a mirror image P_1 corresponding to the 15-nt arm, with AA-ICL2 migrating similarly to the larger product observed for AA-ICL1. Additionally, a previously unobserved minor and nonspecific products (PN) was detected. **(C, D)** Data from panels A and B were plotted and fitted with exponential decay to determine reaction rates with error bars representing standard deviation (SD). However, for both crosslinked and non-crosslinked AA-ICL2 substrates, the 3′ fluorescent label appears to impede the formation of the second product, P_2, as demonstrated in the kinetic data (**C**, red curve).

Supplementary Figure S4: Expression and purification of SXE complex and its mutant forms.

A

Mutation	Primer sequence
mXPF D688A_fw	GCCATGCGTGAGTTTCGGAGCGAGCTCC
mXPF R690S_fw	GACATGTCTGAGTTTCGGAGCGAGCTCCC

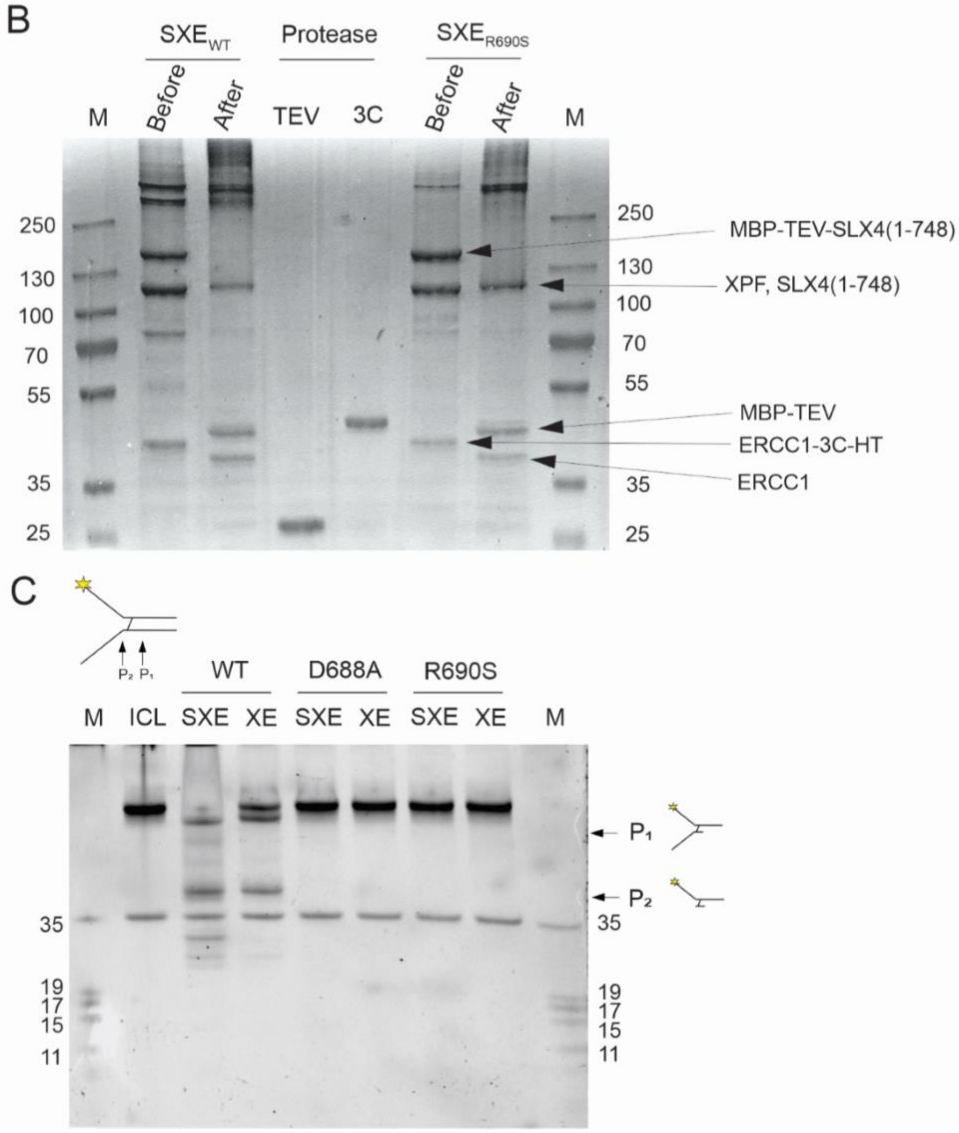

(A) Primers used for mutation. **(B)** Cleavage of fusion tags and purification of final protein complex. **(C)** Enzymatic assay of mutant forms of SXE and XE. Cleavage of AA-ICL after 60 minutes.

Supplementary Figure S5: Graphs with individual data points

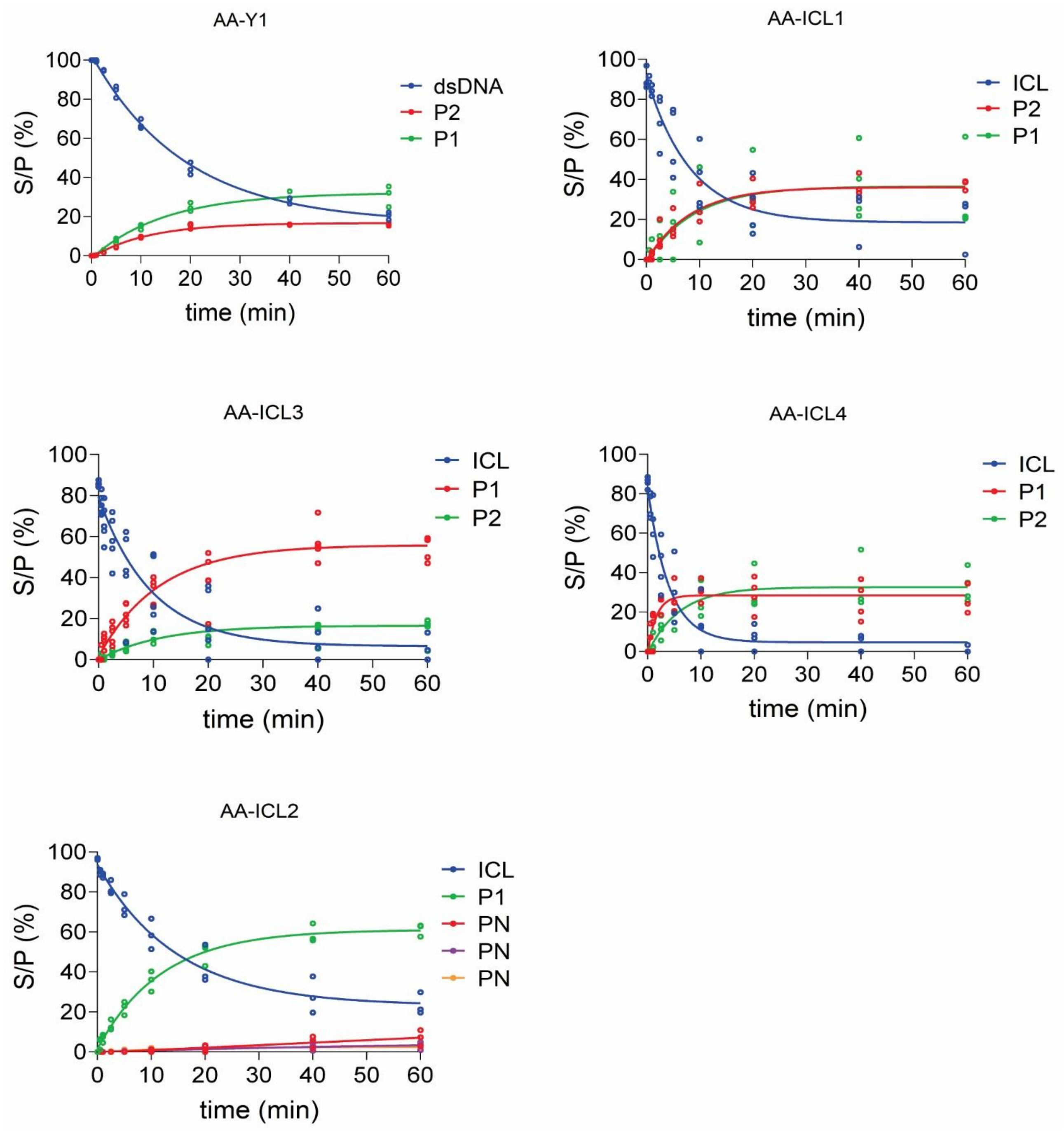

Graphs with individual datapoints from SXE kinetics as shown in Figures 3-4 and S3.

Supplementary Figure S6: Uncropped and unmodified gels

Raw uncropped gel Figure 2B

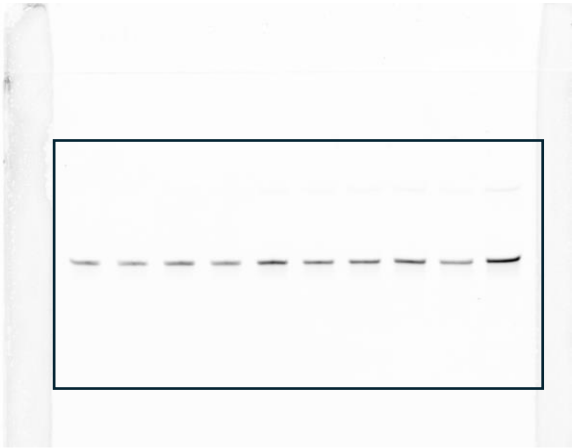

Raw uncropped gel Figure 2D

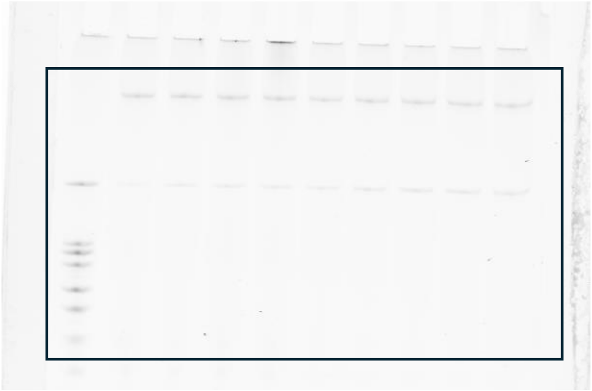

Raw uncropped gel Figure 3B

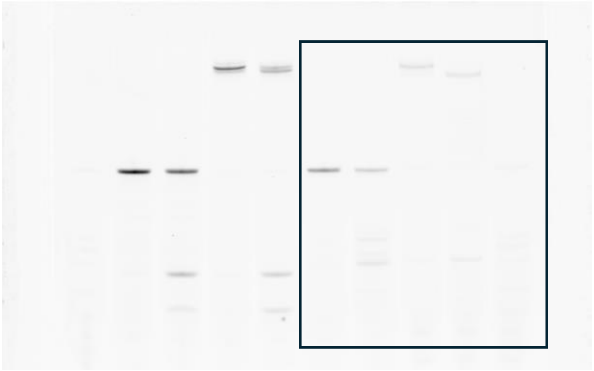

Raw uncropped gel Figure 3C

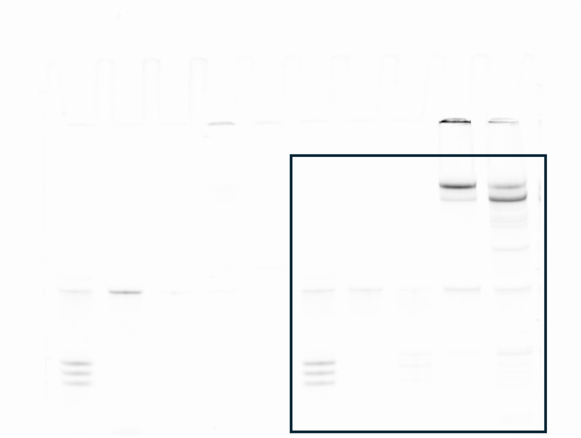

Raw uncropped gel Figure 3D

(left)

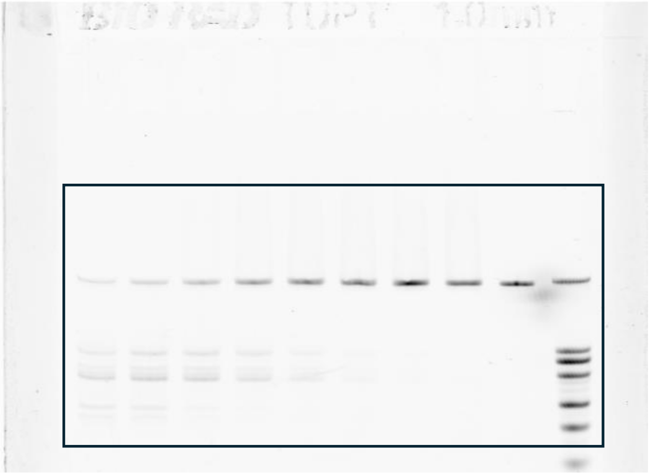

(right)

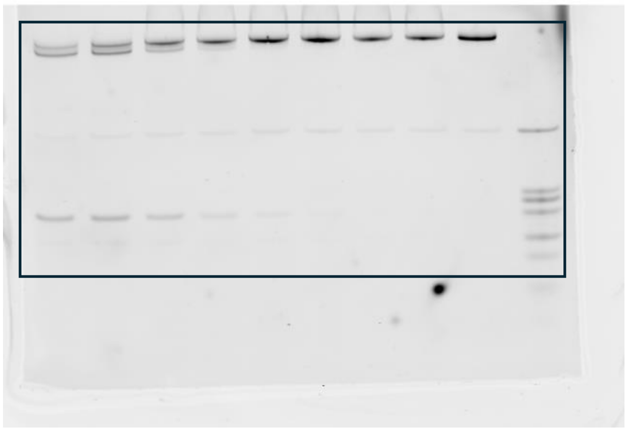

Raw uncropped gel Figure 4B

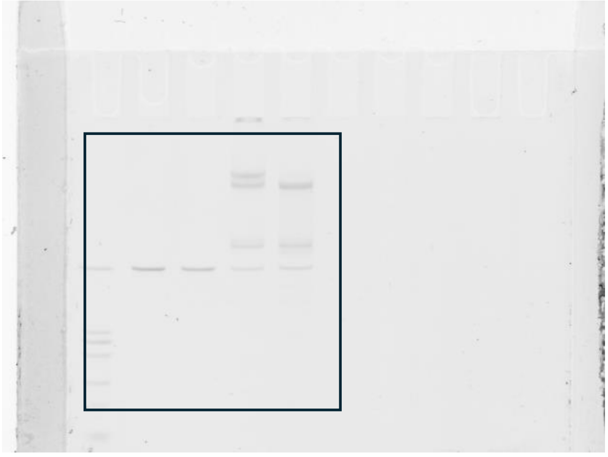

Raw uncropped gel Figure 4D

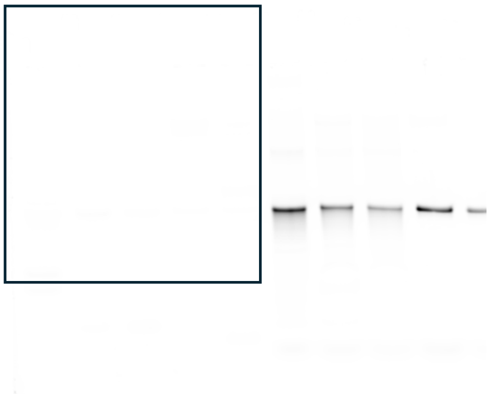

Raw uncropped gel Figure 4C

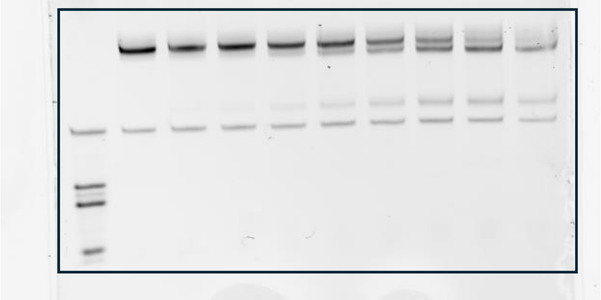

Raw uncropped gel Figure 4E

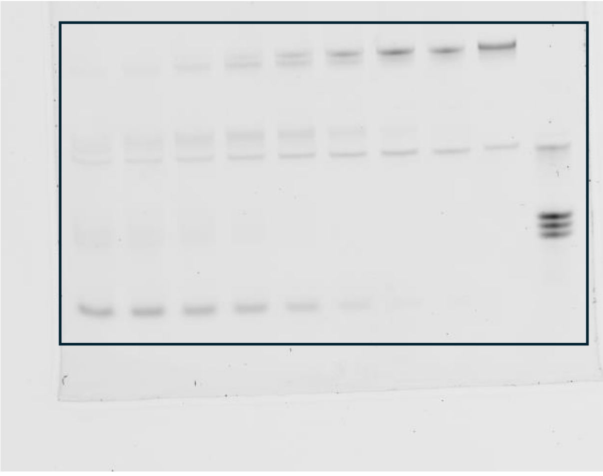

Raw uncropped gel Figure 5B

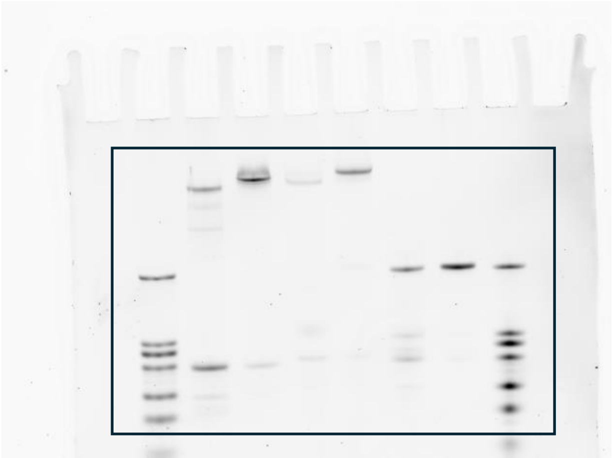

Raw uncropped gel Figure 5C

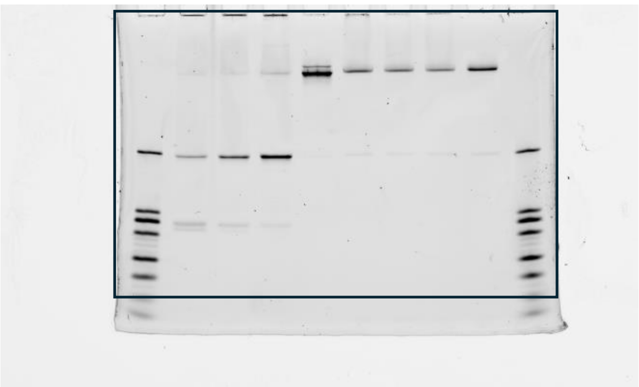

Raw uncropped gel Figure S1B

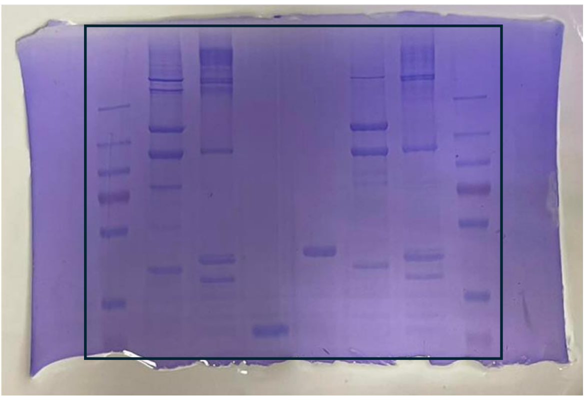

Raw uncropped gel Figure S1C

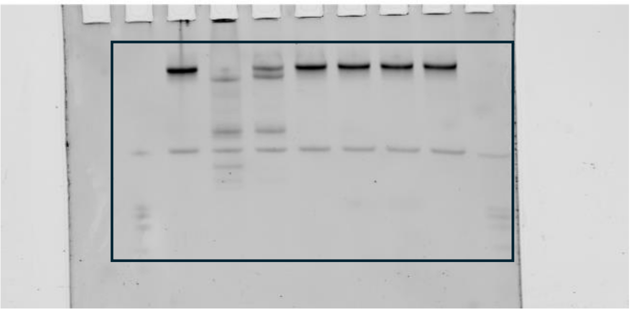

Raw uncropped gel Figure S4A

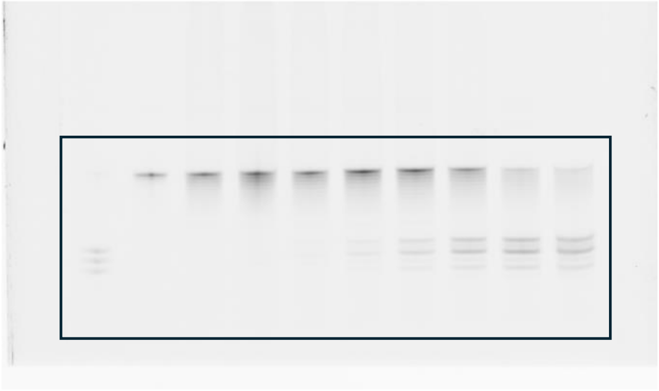

Raw uncropped gel Figure S4B

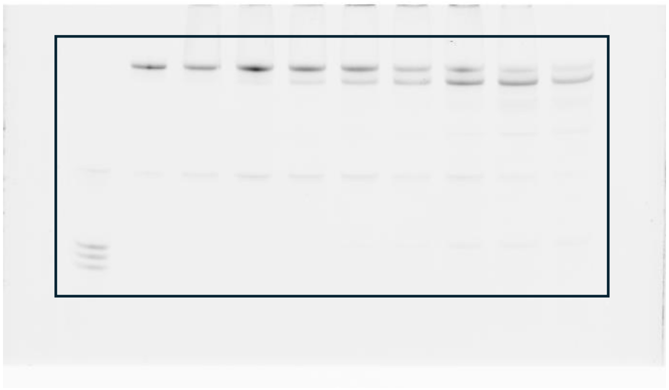

Supplement: Supplementary file 1 — Supplementary Information [file 42003_2025_8769_MOESM1_ESM.pdf]
