# Supplementary material for: Mechanistic insights into alcohol-induced DNA crosslink repair by Slx4-Xpf-Ercc1 nuclease complex in the Fanconi anaemia pathway
Source: Commun Biol. 2025 Sep 26;8:1374. doi: 10.1038/s42003-025-08769-3 (PMC12475018; doi:10.1038/s42003-025-08769-3)
Supplement: Supplementary file 2 — Description of Additional Supplementary Materials [file 42003_2025_8769_MOESM2_ESM.pdf]

Description of Additional Supplementary Files

File name: Supplementary Data 1

Description: Raw data points used for all individual graphs including fitting statistics.
